# Indium 111-labeled J591 anti-PSMA antibody for vascular targeted imaging in progressive solid tumors

**DOI:** 10.1186/s13550-015-0104-4

**Published:** 2015-04-29

**Authors:** Neeta Pandit-Taskar, Joseph A O’Donoghue, Chaitanya R Divgi, Eze A Wills, Lawrence Schwartz, Mithat Gönen, Peter Smith-Jones, Neil H Bander, Howard I Scher, Steven M Larson, Michael J Morris

**Affiliations:** Molecular Imaging and Therapy Service, Department of Radiology, Memorial Sloan Kettering Cancer Center, 1275 York Avenue, New York, NY 10065 USA; Department of Radiology, Weill Medical College of Cornell University, 1300 York Avenue, New York, NY 10065 USA; Department of Medical Physics, Memorial Sloan Kettering Cancer Center, 1275 York Avenue, New York, NY 10065 USA; Genitourinary Oncology Service, Department of Medicine, Memorial Sloan Kettering Cancer Center, 1275 York Avenue, New York, NY 10065 USA; Department of Medicine, Weill Medical College of Cornell University, 1300 York Avenue, New York, NY 10065 USA; Department of Biostatistics, Memorial Sloan Kettering Cancer Center, 1275 York Avenue, New York, NY 10065 USA; Nuclear Medicine Service, Department of Radiology, Columbia Presbyterian Medical Center, 630 West 168th Street, New York, NY 10032 USA

**Keywords:** J591 antibody, Solid tumor, Neovasculature, Lesion detectability, Biodistribution

## Abstract

**Background:**

J591 is a monoclonal antibody that targets the external domain of the prostate-specific membrane antigen (PSMA). Besides prostate cancer cells, it also targets the neovasculature of non-prostate solid tumors. We provide an analysis of the antibody mass-dose dependency of lesion uptake and normal tissue retention, together with an assessment of lesion detectability using ^111^In-J591 imaging, compared with conventional imaging in patients with a variety of solid tumors.

**Methods:**

Twenty patients in six cohorts received fixed amounts (5, 10, 20, 40, 60, and 100 mg) of J591 in a phase I trial. A maximum of four administrations per patient was given, with each administration separated by 3 weeks. All antibody administrations included 370 MBq (10 mCi) of ^111^In labeled to 2 mg of J591 *via* the chelating agent DOTA. Three whole body (WB) gamma camera scans with at least one SPECT scan, along with multiple WB count-rate measurements and blood samples, were obtained for all patients. The effect of escalating antibody mass on lesion uptake and normal tissue retention was evaluated using lesion, liver, serum, and WB residence times and ratios thereof for each treatment cycle. Lesion detectability using ^111^In-J591 imaging was compared to the standard imaging on a lesion-by-lesion basis.

**Results:**

A total of 170 lesions in 20 patients were detected by standard or ^111^In-J591 imaging. ^111^In-J591 targeted both skeletal and soft tissue diseases in all tumor types. ^111^In-J591 imaging identified 74% (20/27) of skeletal lesions, 53% (18/34) of nodes, and 64% (70/109) of other soft tissue/organ lesions. There was increasing ^111^In-J591 uptake in lesions with increasing antibody mass-dose, coupled with decreasing retention in the liver for increments up to 20 mg, and no significant change at higher antibody mass.

**Conclusions:**

Radiolabeled J591 antibody has potential as a targeting agent for solid tumor vasculature and lesion detection. Bone and soft tissue lesions arising from tumors of diverse origin were targeted by the anti-PSMA antibody J591. For the detection of lesions in these tumors by J591 antibody scans, an antibody mass of 20 mg is adequate. The optimal time of imaging is 5 to 7 days post-injection.

## Background

Prostate-specific membrane antigen (PSMA) is a tumor marker associated with prostate cancer. It is a 100-kDa transmembrane glycoprotein found on prostate epithelial cells including both benign and malignant prostatic tissues [1-4]. While PSMA expression appears to be greatest in prostate adenocarcinoma, primary tumor, and nodal metastasis [5,6], it is also present in the neovasculature of solid tumors [7-12]. Most commonly expressing tumors include renal, lung, gastric, colon, and breast [7,13]. It has been shown that the expression is associated with the endothelium of the neovasculature of these tumors [7]. Tissue microarray analysis of the renal tumors showed PSMA expression and positive PSMA staining in tumor-associated vasculature in 76.2% of CCRCC, 31.2% of chromophobe RCC, 52.6% of oncocytoma, and 21.4% of transitional cell carcinoma (TCC) [12]. J591 is a humanized monoclonal IgG1 antibody that targets the external domain of PSMA [14-16].

Pilot studies to evaluate safety and biodistribution in patients with solid tumors have shown that J591 is safe to administer, localizes to bone and soft tissue metastatic sites, and exhibits serum pharmacokinetics and hepatic uptake that is dependent on the mass-dose of the antibody [17-19]. Detection of the lesions appears to be dependent on the antibody mass and time of imaging [20], while the determination of antibody mass dependency helps to establish optimal doses for immunotherapy and radioimmunotherapy [21].

An initial analysis of the use of ^111^In-J591 as a vascular tumor targeting agent in a phase I mass-dose-escalation study has been previously reported [18,22]. It was observed that the clearance rate of antibody from serum was inversely related to antibody mass-dose, that liver uptake of antibody was also dependent on antibody mass-dose with greater proportional uptakes seen for lower mass-doses, and that liver saturation appeared to occur by 60 mg, based on point estimates of proportional hepatic uptake. A preliminary analysis of lesion targeting showed that 17 of 18 (94%) patients with soft tissue disease on standard scans showed uptake in the soft tissues on antibody scans, as did 6 of 6 patients with bone disease. We now report a more detailed analysis for lesion detectability with ^111^In-J591 on a lesion-by-lesion basis in patients with a variety of solid tumors. The lesion detection and residence times are evaluated in relation to the whole body (WB), serum, and liver ^111^In-J591 residence times.

## Methods

### Clinical study

Patients with histologically proven, advanced non-prostate solid tumors with evidence of disease progression were eligible. The clinical protocol was approved by the Institutional Review Board of Memorial Sloan Kettering Cancer Center, and all patients signed written informed consent forms [18]. Each patient received up to a maximum of four administrations of an identical mass-dose of J591 antibody, each separated by 3 weeks. The mass-dose of antibody was increased between cohorts (each of three patients) starting at 5 mg and increasing through 10, 20, 40, and 60 mg to a maximum of 100 mg. Every antibody infusion included a fixed amount of radiolabeled antibody ^111^In-DOTA-J591 (2 mg of J591 labeled with 370 MBq [10 mCi] of ^111^In). The patients did not receive any other concurrent therapy during the study period, including between the infusion cycles.

### Imaging

Anterior and posterior WB planar scans were acquired on a Philips dual-head gamma camera (Philips Inc., Andover, MA, USA), using dual-energy acquisition centered at 171 and 245 keV with 20% windows. Following each antibody administration, patients underwent at least three WB scans, the first obtained within 2 to 4 h of administration, followed by at least two additional scans between 24 and 196 h. Patients were also imaged by single-photon emission computerized tomography (SPECT) after each antibody administration. SPECT images were generated using iterative reconstruction and attenuation correction.

### WB and serum measurements

WB clearance was assessed by serial measurements of count-rate using a 12.7-cm (5 in.)-thick sodium iodide NaI (Tl) scintillation probe. Duplicate anterior and posterior measurements were made at fixed geometry, and background-corrected geometric mean values were used for clearance curve fitting. Probe measurements were made immediately post-administration and subsequently for up to 7 days following antibody administration. An ^111^In standard of known activity was counted contemporaneously. The median number of WB count-rate measurements was 6 (range 4 to 7). The count rates were normalized to the value immediately post-administration (taken as 100%) to yield relative retained activities (in %).

A median of 10 (range 8 to 10) venous blood samples (approx 5 ml) were drawn at nominal times of 5, 15, 30, 60, and 120 min and on multiple occasions up to 7 days following administration. Aliquots (500 μl) of serum were counted in duplicate in a NaI (Tl) gamma well-type detector (Wallac Wizard 1480 automatic gamma counter, PerkinElmer Inc., Waltham, MA, USA) calibrated for ^111^In, with the net count rates converted to activities and the results expressed in percentage of the injected dose per unit volume (%ID/l).

### Derivation of kinetic parameters

A mono-exponential function was fitted to the WB data, and both mono- and bi-exponential functions were fitted to the serum data using the SAAM II software application [23]. Biological and effective clearance rates and corresponding half-times for WB and serum were estimated for each treatment cycle. Subsequently, cumulative activity per unit of administered activity (also known as residence time), *τ*, was calculated for WB (in h) and serum (in h/l) according to the formula *τ* = *Ã*/*A*_0_, where *Ã* equals the cumulated activity (derived by integration of the activity-time curve) and *A*_0_ is the administered activity.

### Determination of uptake in liver and lesions and comparative metrics

Regions of interest (ROI) were drawn on anterior and posterior gamma camera images to encompass the whole liver, up to two index lesions, and normal tissue background. Typically, ROI were drawn on the latest images (where lesions were generally most clearly seen) then copied and pasted to the other images. In all, a total of 19 liver ROI and 23 lesion ROI (in 14 patients) were examined. The background-corrected, geometric mean count-rate was used as the primary metric for image-based uptake analysis. Areas under the count-rate-time curve (AUC) for WB, liver, and lesions were estimated by trapezoidal integration. The contribution of the area under the terminal portion of the clearance curve was estimated by extrapolation using the apparent terminal clearance rate or physical decay, whichever rate was shorter. Subsequently, the relative AUC for liver and lesions was calculated as fractions of the image-derived AUC values for WB and converted to cumulative activities per unit of administered activity (residence times) using the probe-derived WB residence time estimates.

Comparative metrics were derived based on the ratios of residence times; in particular, lesion to liver, lesion to WB, and liver to WB. These were used to compare the different mass cohorts. In addition, a normalized lesion-to-liver ratio was used in an effort to investigate trends with increasing cycle numbers. This metric was constructed as follows: for each lesion at each imaging time, a lesion-to-liver count-rate ratio was derived; these ratios were then normalized to the first non-day-of-administration image for the first cycle. Finally, the average values of these ratios were calculated for each cycle, excluding any images acquired on the day of administration. The advantage of this metric was that it eliminated the lesion size dependency of the lesion-to-liver ratio.

### Image interpretation and lesion detection

All images were reviewed for tumor targeting by two nuclear medicine physicians, blinded to other conventional imaging results. For each patient, anterior and posterior WB images and SPECT images were visually analyzed. Sites of abnormal uptake were defined as those that were not within the physiologic distribution of the antibody (blood pool, liver, mild diffuse spleen, and renal and GI activity) and that appeared more focal and intense. All foci of abnormal uptake had to be clearly delineated in two views and visually greater than the adjacent normal background activity. All visualized areas of increased uptake were graded on a scale of 1 to 5 (1 = negative, 2 = probably negative, 3 = equivocal, 4 = probably positive, and 5 = definitely positive), based on a visual comparison with background activity in a normal adjacent region. Grade 5 indicates very intense activity (similar to liver); grade 4 signifies a definite increase in activity but less than that of the liver; grade 3 is equivocal to uptake in adjacent normal background; grade 2 is less than adjacent normal background uptake; and grade 1 is no uptake. The anatomic locations of all such areas were recorded.

Baseline CT scans and Tc-99m MDP bone scans were reviewed for lesions by a radiologist who was blinded to the results of the antibody scans. All lesions detected by each modality were recorded separately, and the lesions detected by antibody scans were compared to those detected by CT or bone scan. The CT and bone scans were performed clinically within a month of the antibody infusion and imaging. The CT scans and bone scans were independently read by separate radiologists. Detected lesions were noted and characterized on a similar (1 to 5) scale for possibility of disease. CT scans were read per RECIST 1.1 criteria, soft tissue disease was measured per RECIST 1.1, and bone lesions were measured if they were lytic with soft tissue component.

### Statistics

Parameter estimates are quoted in terms of mean values and associated standard errors. For lesion detection, detection rates of the three modalities were compared using McNemar’s test. All analyses took into account the clustering that resulted from multiple lesions per patient using the methods described by Gonen et al. [24].

## Results

### Patients

A total of 20 patients (12 male and 8 female) were treated in 6 cohorts starting at a dose of 5 mg up to 100 mg of J591. The median age of the patients was 65 (range of 39 to 80). The distribution of tumors included melanoma (*n* = 5) and cancers of the kidney (*n* = 5), colon (*n* = 2), head and neck (*n* = 2), TCC of renal pelvis (*n* = 2), stomach (*n* = 1), bladder (*n* = 1), breast (*n* = 1), and liver (*n* = 1). For the mass-dose levels of 5, 20, 40, and 60 mg, three patients were included in each cohort. Four patients were treated in cohorts 2 (10 mg) and 6 (100 mg) due to the need for replacement of patients who could not continue for medical reasons unrelated to the antibody infusions following the first antibody administration. Patients received a median of 2 cycles (range 1 to 4) with 16 patients receiving 2 or more cycles. The injected activity range was 9.3 to 10.4 mCi (mean 9.9 mCi of ^111^In). Lesion detection analysis was performed on all 20 patients, while mass escalation analysis was performed only on those who received more than 1 cycle.

### Uptake and retention in WB, serum, liver, and lesions

**Figure 1:**
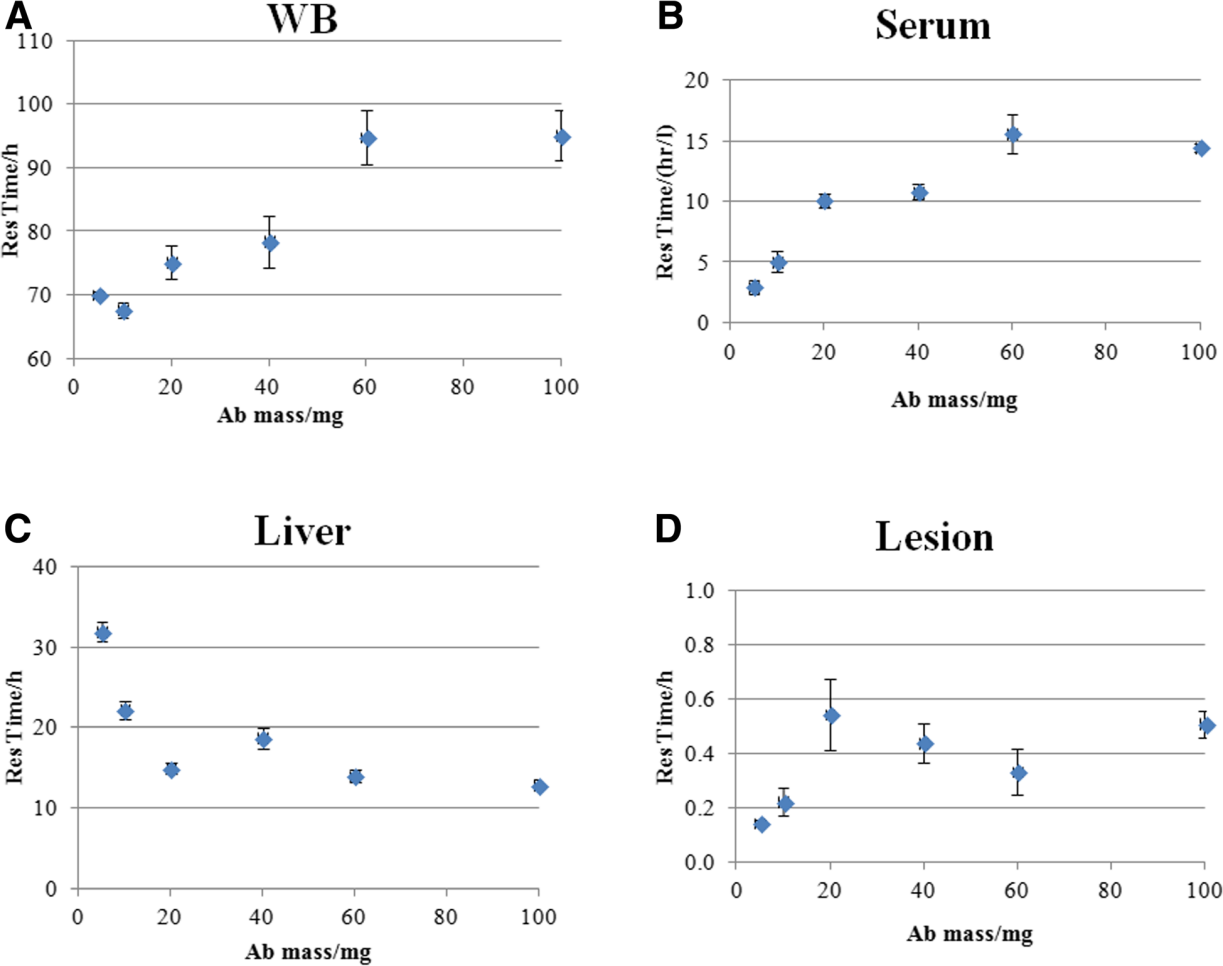
Residence times with increasing antibody mass in whole body (WB) **(A)**, serum **(B)**, liver **(C)**, and lesion **(D)**. Values are mean parameter estimates for all available cycles for the patients within a cohort. Error bars represent standard errors.

Figure 1A,B,C,D illustrates graphically the antibody mass dependency of residence time estimates for WB, serum, liver, and index lesions (numerical values are also provided in Table 1). There was little difference between mono- and bi-exponential curve-based serum residence times; the values shown are for mono-exponential clearance. These data show increasing retention in WB, serum, and lesions with increasing antibody mass, coupled with decreasing retention in liver. For lesions and liver, there is little evidence of mass dependency for antibody masses greater than 20 mg, whereas for WB and serum, mass dependency appears to extend up to the 60 mg level.

**Table 1:** **Summary of kinetic/uptake data**

**Antibody mass (mg)**	**5**	**10**	**20**	**40**	**60**	**100**
WB residence time (h)	70.0 ± 0.6	67.6 ± 1.2	75.1 ± 2.7	78.4 ± 4.1	94.8 ± 4.3	95.1 ± 3.9
Serum residence time (h/l)	2.9 ± 0.6	5.0 ± 0.8	10.1 ± 0.6	10.8 ± 0.6	15.6 ± 1.6	14.5 ± 0.3
Liver residence time (h)	31.9 ± 1.1	22.2 ± 1.1	14.9 ± 0.7	18.7 ± 1.3	14.0 ± 0.7	12.9 ± 0.6
Lesion residence time (h)	0.14 ± 0.01	0.22 ± 0.05	0.54 ± 0.13	0.44 ± 0.07	0.33 ± 0.08	0.51 ± 0.05

Values are mean parameter estimates for each administered mass of hu-J591. Quoted uncertainties are the standard error of the mean.

**Figure 2:**
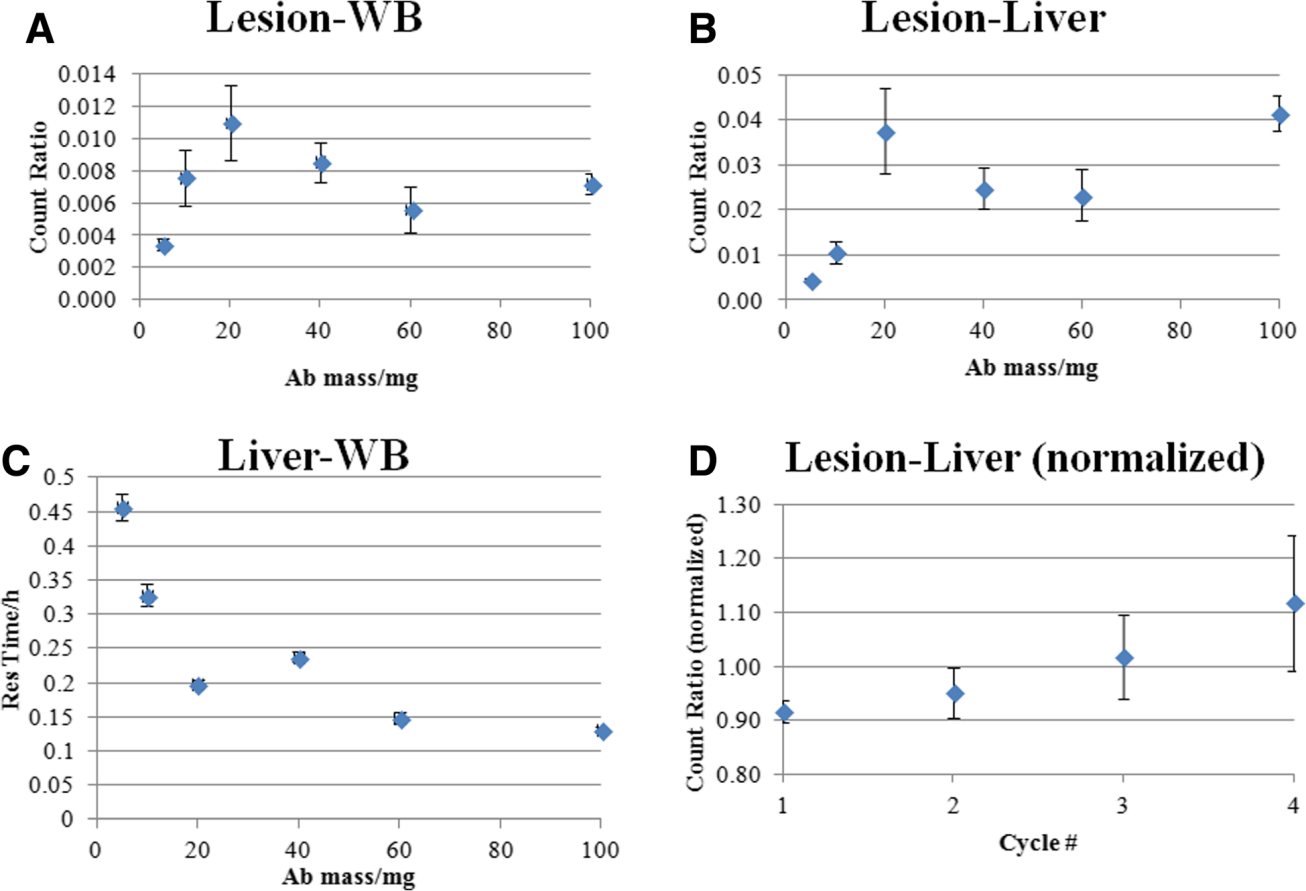
Lesion uptake ratios. Lesion-to-WB ratio **(A)**, lesion-to-liver ratio **(B)**, liver-to-WB ratio **(C)**, and normalized lesion-to-liver ratio **(D)**. Values are mean parameter estimates for all available cycles for the patients within a cohort. Error bars represent standard errors.

Figure 2A,B,C,D shows a summary of the comparative metrics of uptake and retention in lesions, liver, and WB as a function of antibody mass. Similar to the lesion residence times, the general trends indicate an increase in comparative lesion uptake with antibody mass of up to 20 mg with no obvious increase thereafter (Figure 2A,B), coupled with a decrease in comparative liver uptake of up to 60 mg (Figure 2C). There was a slight (statistically non-significant) trend of an increase in normalized lesion-to-liver ratio trend with higher cycle numbers (Figure 2D).

### Image interpretation and lesion detection

Twenty patients with a variety of solid tumors including melanoma (*n* = 5) and cancers of the kidney (*n* = 5), colon (*n* = 2), head and neck (*n* = 2), TCC of renal pelvis (*n* = 2), stomach (*n* = 1), bladder (*n* = 1), breast (*n* = 1), and liver (*n* = 1) were included in the study. For the mass-dose levels of 5, 20, 40, and 60 mg, three patients were included in each cohort. Four patients were treated in cohorts 2 (10 mg) and 6 (100 mg) due to the need to replace patients who could not continue for medical reasons (unrelated to the antibody infusions) after the first antibody administration. Patients received a median of 2 cycles (range 1 to 4), with 16 patients receiving 2 or more cycles.

**Figure 3:**
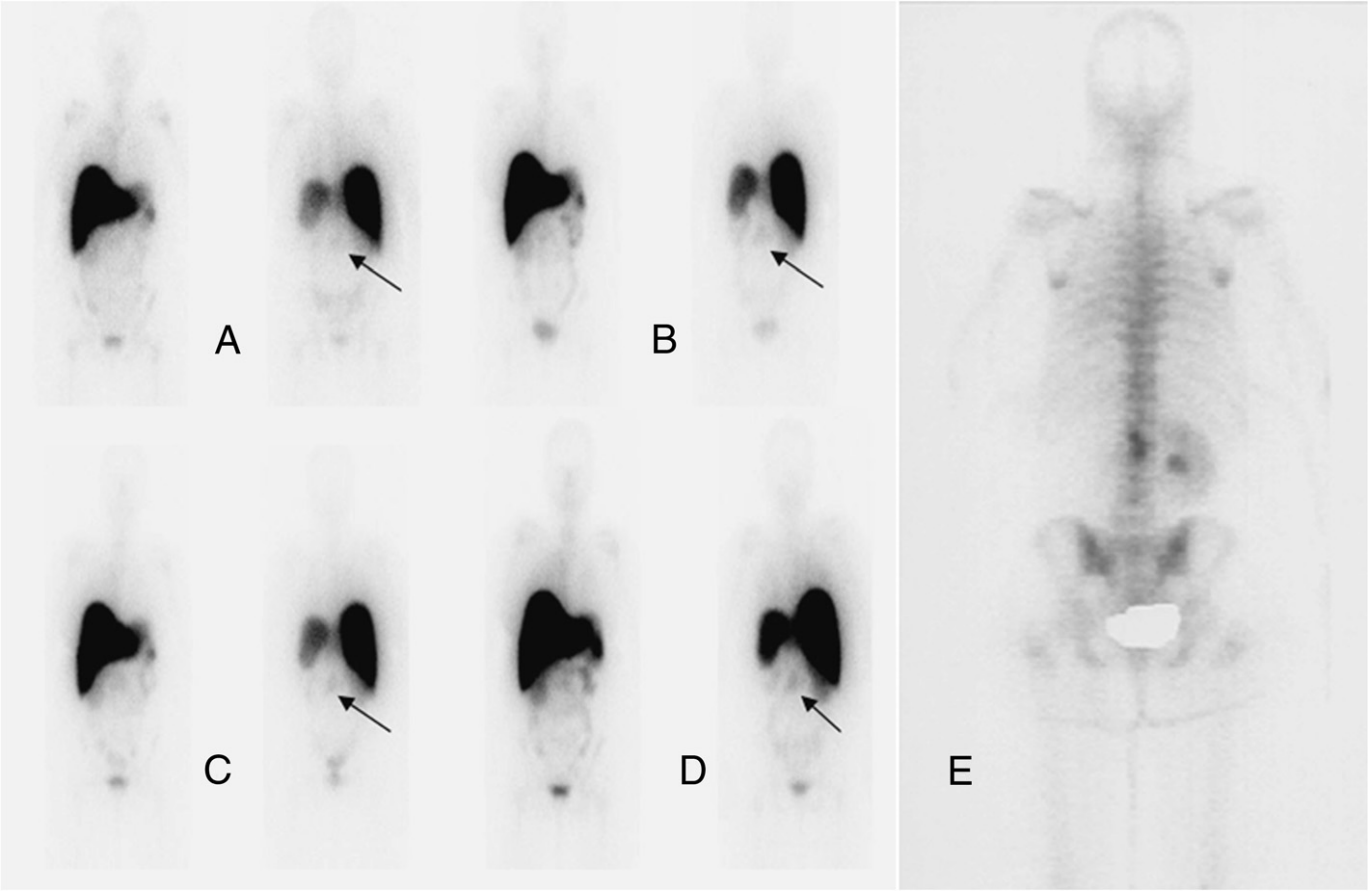
Patient with metastatic renal cell carcinoma. Received 5 mg of ^111^In hu-J591 for 4 cycles. Anterior and posterior WB images from cycles 1 to 4 are shown from left to right **(A-D)**. Uptake in the lumbar lesion **(E)** (bone scan) is seen in all images from cycles 1 to 4 **(A-D)** with most prominent uptake in the last cycle **(D)**.

**Figure 4:**
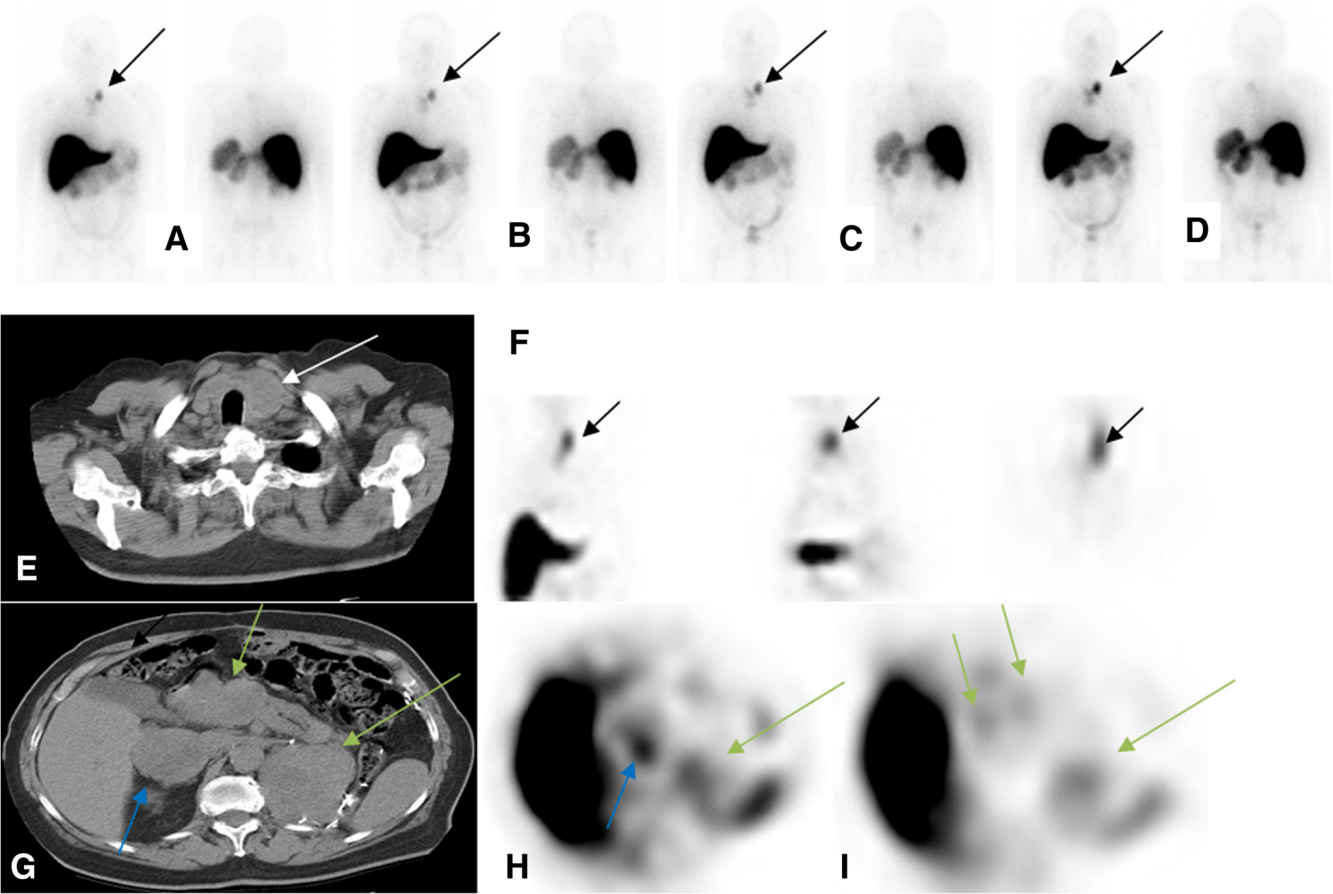
Patient with metastatic renal cell carcinoma post left nephrectomy. Patient received 5 mg of ^111^In hu-J591. Delayed WB images (day 6 or 7; cycles 1 to 4) show uptake in left neck (**A** to **D**, respectively; black arrows) corresponding to the left paratracheal nodal mass seen on CT image **(E)** (white arrow). This is also seen in the coronal, sagittal, and axial SPECT images (respectively in **(F)**). Uptake was also seen corresponding to the right adrenal mass (blue arrow), pancreatic head mass (short green arrows), and left renal bed mass (long green arrow) (CT image **(G)** and axial SPECT images **(H)** and **(I)**).

All antibody scans were positive for either bone or soft tissue lesions (Figures 3 and 4). A total of 170 lesions, including 27 bone lesions, 34 nodal lesions, and 109 lesions in non-nodal soft tissue or organs were detected by any imaging modality including conventional imaging (CT or bone scan) or antibody imaging (Tables 2 and 3). The organs included the lung, liver, renal fossa, spleen, adrenal, and bladder. Other soft tissue lesion sites included the skin or subcutaneous tissue.

**Table 2:** **Lesion distribution and detection in patients**

	**Number**
Patients	
Total	20
Bone metastasis	9
Nodal metastasis	14
Other organ metastasis	7
Lung	11
Liver	6
Lesions	
Total lesions	170
Bone	27
L. node and organs (soft tissue)	143
Concordance: bone lesions	
CT or bone scan positive and antibody scan (Ab) positive	20
CT or bone scan positive, Ab scan negative	7
CT and bone scan negative, Ab scan positive	0
Concordance: nodal lesions	
CT scan and antibody scan (Ab) positive	18
CT scan positive, Ab scan negative	16
CT scan negative, Ab scan positive	0
Concordance: soft tissue lesions	
CT scan and antibody scan (Ab) positive	69
CT scan positive, Ab scan negative	39
CT scan negative, Ab scan positive	1

**Table 3:** **Lesion detection by modality**

**Primary tumor**			**Skeletal lesions seen**			**Soft tissue lesions or organ involvement**			**Nodes seen**		
	**Ab dose (mg)**	**No. of cycles**	**Total**	**CT scan or BS**	**Antibody scan**	**Total**	**CT scan**	**Antibody scan**	**Total**	**CT scan**	**Antibody scan**
Melanoma	5	2	0	0	0	3	3	3	1	1	0
Renal cell	5	4	3	3	3	6	6	4	3	3	2
Renal cell	5	4	2	2	2	9	9	4	1	1	0
Hepatocellular	10	1	0	0	0	6	6	4	0	0	0
Melanoma	10	2	4	4	0	12	12	7	3	3	2
Melanoma	10	2	1	1	0	5	5	5	3	3	3
Renal cell	10	4	0	0	0	8	7	4	2	2	1
Adenoca colon	20	2	3	3	2	12	12	10	3	3	0
Melanoma	20	2	5	5	5	8	8	2	0	0	0
SCC hypoph	20	2	0	0	0	2	2	2	1	1	1
TCC renal pelvis	20	4	0	0	0	2	2	2	1	1	0
Adenoca colon	40	1	0	0	0	2	2	1	0	0	0
TCC renal pelvis	40	2	0	0	0	1	1	1	3	3	2
Melanoma	60	2	1	1	1	8	8	3	3	3	3
Renal cell	60	1	0	0	0	5	5	4	2	2	2
Renal cell	60	4	0	0	0	6	6	3	0	0	0
Adenoca gastric	100	4	0	0	0	5	5	5	3	3	0
Breast	100	1	7	7	6	0	0	0	0	0	0
SCC tongue	100	2	1	1	1	3	3	3	0	0	0
TCC-bladder	100	2	0	0	0	6	6	3	5	5	2
			27	27^a^	20	109	108	70	34	34	18

^a^Total bone lesions detected by CT only: 13. Adenoca, adenocarcinoma; hypoph, hypopharynx; SCC, squamous cell carcinoma; TCC, transitional cell carcinoma.

Out of 27 bone lesions detected by CT or bone scan, 20 were identified by antibody scan (Tables 2 and 3). CT alone detected 13/27 (48%) of bone lesions. There were 7 (26%) bony lesions seen on bone or CT scans that were not seen on antibody imaging, and 14 lesions seen on bone scan were not detected by CT scan, while the bone scan detected all lesions. Those bone lesions not seen with antibody imaging included sub-centimeter rib lesions located anterolaterally in the chest and small lesions in the transverse process close to costovertebral junctions in the thoracic vertebrae. In one patient, a pubic symphysis lesion was difficult to detect due to a combination of overlap with bladder activity and small lesion size. There was a statistically significant difference for the detection of bony lesions between CT and antibody imaging (adjusted McNemar’s chi square *P* = 0.01), with antibody imaging identifying significantly more lesions than CT.

Out of 34 nodal lesions detected by CT, antibody imaging identified 18 (53%). Detection was mainly limited by size (generally limited below 2 cm) and/or proximity to the mediastinal blood pool or large vessels in the abdomen and pelvic regions. For non-nodal soft tissue lesions, a total of 109 lesions were seen on either CT scan or antibody scan (Table 2); CT detected 108 (99%) and antibody detected 70 (64%) of the lesions. There were 69 lesions seen concurrently on CT and antibody imaging, while in one case, the antibody scan identified a soft tissue lesion not seen on CT, which was confirmed as a true-positive lesion on follow-up CT scan performed within 1 month after the antibody scan.

**Figure 5:**
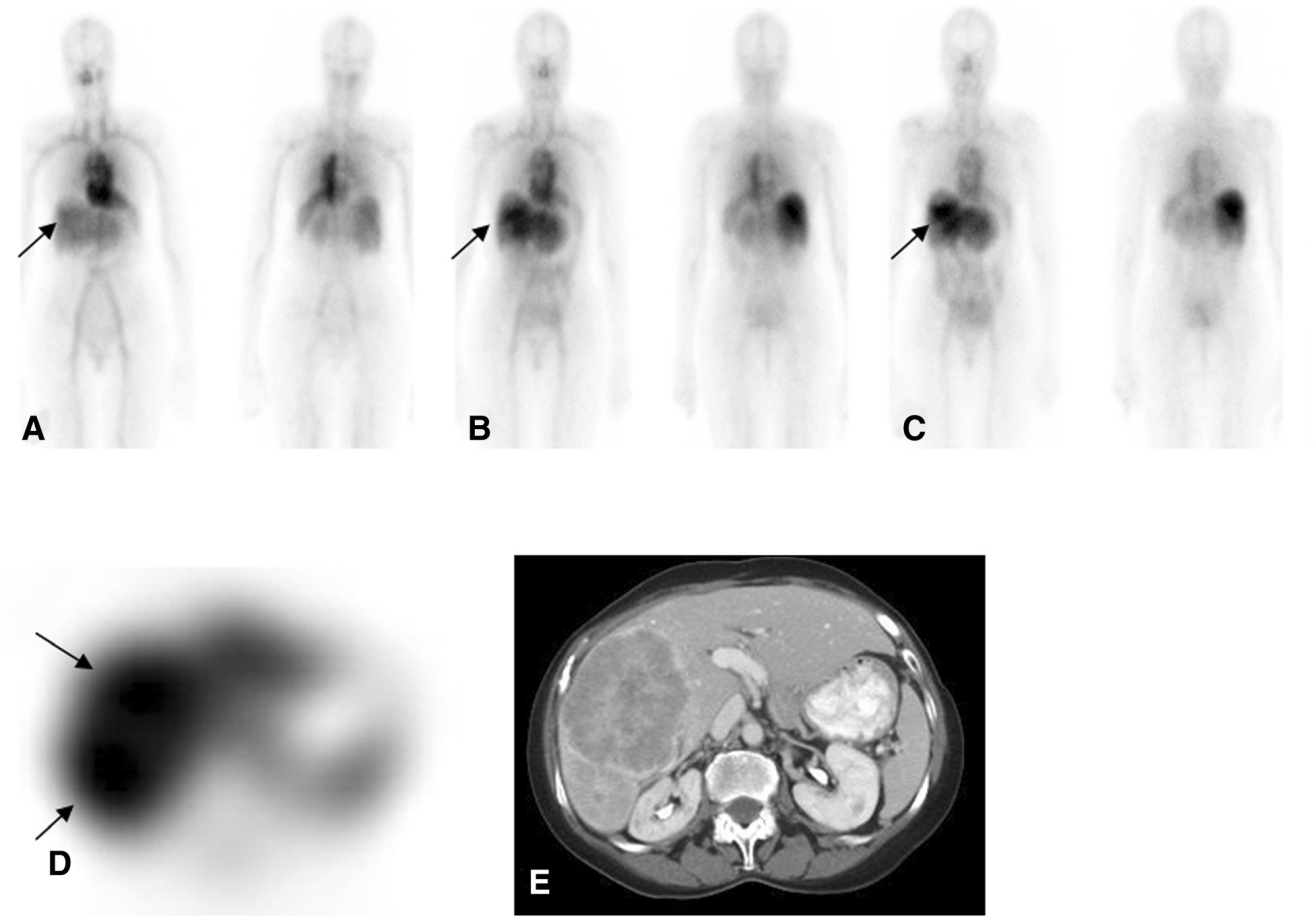
Female with metastatic hepatocellular carcinoma. Anterior and posterior WB images from day 0, day 3, and day 6 (**(A-C)**, respectively) show heterogeneous activity in the liver steadily increasing in later images **(C)**, more clearly seen on SPECT **(D)**, corresponding to the progressive liver disease seen on CT images **(E)**.

Soft tissue lesions not seen with antibody imaging included small lung lesions; of 25 such lesions, 18 were approximately 5 mm in size, with 7 between 1 and 1.5 cm. In addition, the lung lesions located close to the heart or mediastinum tended to be obscured by nearby activity and image noise. Ten lesions in the liver were not detected, probably due to high levels of antibody uptake in normal liver. Due to this physiologic uptake, liver lesions were likely to be missed; although in six patients, liver lesions (mainly larger lesions of >2.5 cm in size) were detected by antibody imaging (Figure 5). Other missed soft tissue lesions included a splenic lesion, a metastatic lesion in an ovary, and two small subcutaneous lesions. Fourteen patients had nodal involvement, while lung and liver involvement was seen in eleven and six patients, respectively. Antibody imaging was positive for other organ involvement in seven patients, including kidney lesions or renal bed recurrence, adrenal bed disease, the pancreas, spleen, bladder, skin, and subcutaneous lesions.

**Figure 6:**
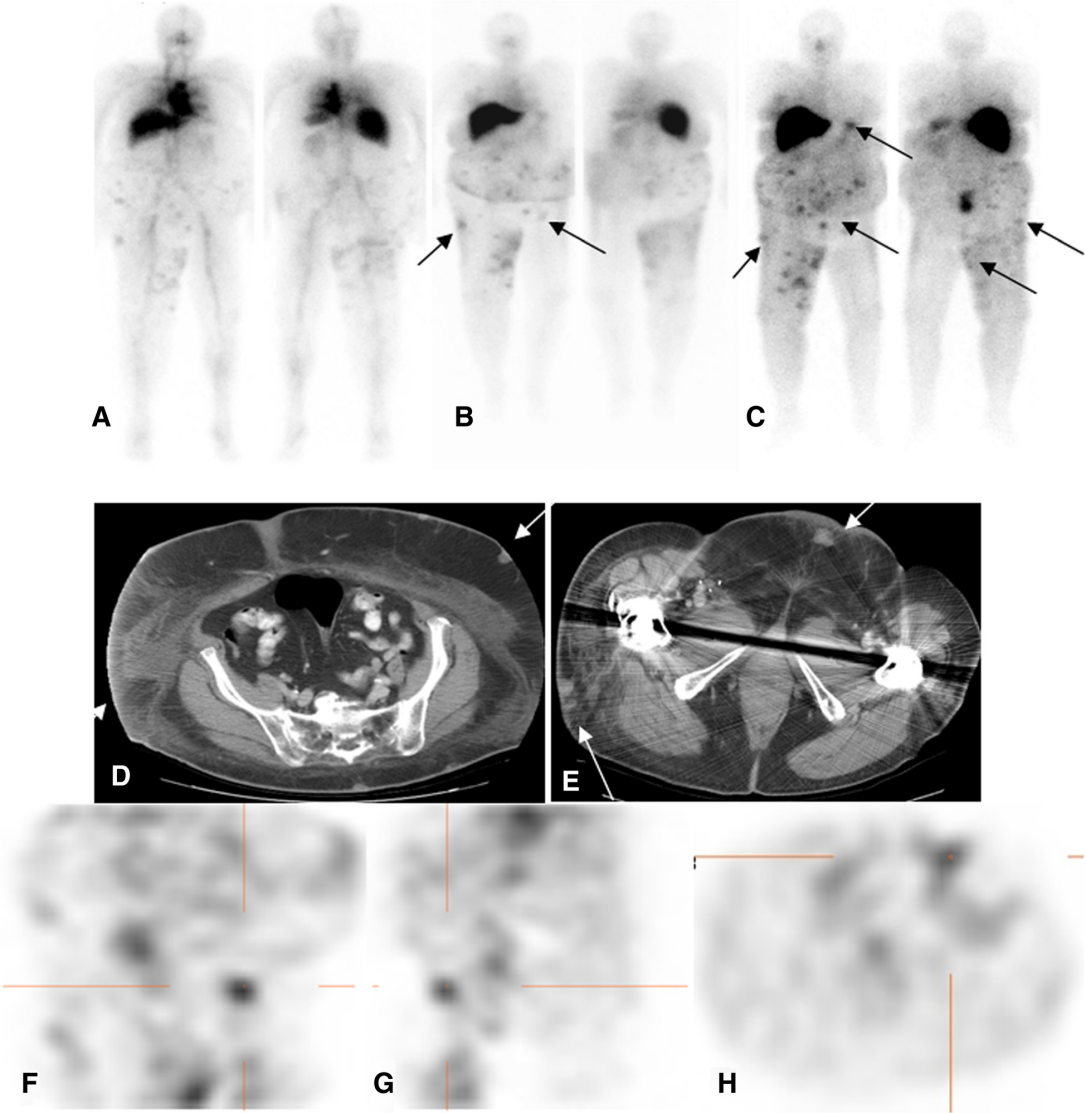
Patient with metastatic melanoma. Treated with 20 mg hu-J591 doses. Day 1, day 4, and day 7 images **(A-C)** after treatment cycle 2 show multiple foci of uptake in the skin and subcutaneous lesions **(D, E)** most prominent in the abdominal wall and right thigh. Uptake also localized focally on SPECT coronal, sagittal, and axial images **(F-H)** along known lesions.

**Figure 7:**
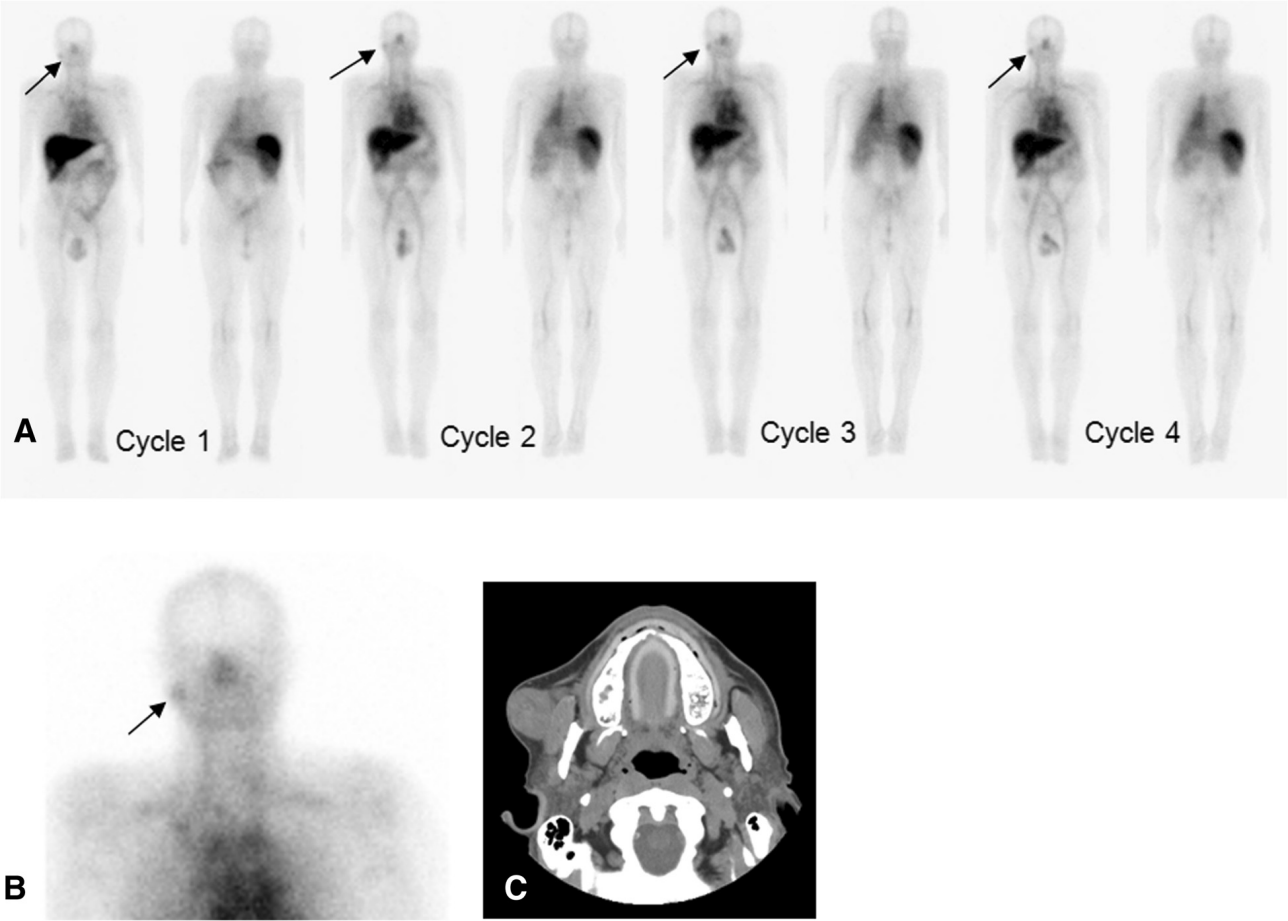
Patient with renal cell carcinoma. Injected with a total of 60 mg of antibody each for 4 cycles. Anterior and posterior WB images from day 4 imaging are shown corresponding to cycles 1 to 4 **(A)** (from left to right). Images show physiologic uptake in liver, blood pool in heart and vasculature, and spleen. Focal activity is seen in the region of right jaw (arrows) **(A,B)**, which corresponds to soft tissue metastasis in right cheek overlying the masseter muscle, confirmed on a follow-up CT scan **(C)**.

Based on a 5-point scoring scale for the detection of lesions, more lesions (>50%) were considered true lesions in the later scans (5 to 7 days after each infusion) than in scans performed earlier (Figures 5 and 6). In addition, lesions were more prominently seen on scans obtained with later infusions for patients with multiple cycles of infusion (cycle 3 or 4) (Figure 7). However, patient numbers were limited for such cases, as not all patients completed 4 cycles of treatment.

## Discussion

We have previously described the feasibility of imaging vascular solid tumors using antibody J591 and its serum pharmacokinetics [18]. The current report describes a detailed analysis of the lesion uptake and targeting based on the full kinetic profiles of ^111^In-J591 using residence times in WB, liver, serum, and index lesions. This analysis showed no significant decrease in liver uptake beyond 20 mg and that the decrease in liver-to-WB ratio can be ascribed to an increase in WB retention, which continues up to an antibody mass of at least 60 mg. The observation that the lesion residence times and lesion-to-liver and lesion-to-WB ratios did not increase for antibody masses greater than 20 mg implies that for detection of vascular lesions by J591 antibody scans, an antibody mass of 20 mg is adequate.

This is the first detailed analysis of lesion-by-lesion targeting of ^111^In-J591 vascular targeted imaging *versus* conventional imaging in solid tumors. Lesion targeting with ^111^In-J591 was seen in all patients and all types of solid tumors imaged in this study. ^111^In-J591 had good detection rates for skeletal lesions (20/27). For bone lesions, the overall sensitivity of antibody scanning was higher than CT alone (74% *vs.* 48%) and it detected more lesions (*n* = 7), including those in the ribs, vertebrae, and proximal femora that were true positives based on visualization and progression on follow-up imaging, including bone scans or CT scans. The comparative numbers with bone scan and CT combined were lower; however, the specificity of targeted imaging of lesions as compared to BS may be a benefit. The current study is limited due to small numbers. Targeting of nodal and soft tissue disease was also seen in all tumor types, although detection rates were lower than for bone lesions. Overall, the positivity for nodal disease was 53% (18/34), with a suggestion of relatively greater targeting to more vascular tumors such as melanoma or renal cell carcinoma where respectively, 86% and 66% of nodal lesions were seen (Table 4); however, these numbers were limited. The majority of soft tissue lesions were non-nodal soft tissue disease totaling 109 lesions, which involved organs, post-surgical bed of resected primaries or skin, and subcutaneous tissue. ^111^In-J591 detected 64% (70/109) of these lesions.

**Table 4:** **Lesions detected by** ^**111**^ **In-J591 imaging out of the total detected**

**Number of patients**	**Tumor type**	**Bone lesions**	**Organ lesions**	**Nodal lesions**
3	Colon/Gastric	2/3	16/19	0/6
3	Bladder/Renal TCC	0/0	6/9	4/9
5	Melanoma	6/11	20/36	8/10
1	Breast	6/7	0/0	0/0
1	Liver-HCC	0/0	4/6	0/0
5	Renal cell	5/5	19/34	5/8
2	Head & neck SCC	1/1	5/5	1/1

TCC, transitional cell carcinoma; HCC, hepatocellular carcinoma; SCC squamous cell carcinoma.

In all cases, the primary reasons for failing to detect lesions on antibody scan included small lesion size and locally high normal tissue background due either to proximity of the heart or major vessels (lungs) or physiological uptake (liver). In this study, antibody imaging was performed using planar scintigraphy and SPECT only and was thus particularly susceptible to uncertainties associated with overlapping activities and structural misidentification. We anticipated that lesion detection and localization would be superior with SPECT/CT imaging or PET/CT imaging. Another important issue was the antibody mass-dose dependency of lesion detection. The analysis suggests that lesion detectability is likely to be optimal for antibody mass-doses of equal to or greater than 20 mg. However, due to the small number of patients, the range of antibody mass-doses investigated, and the diversity of the clinical population, the analysis was limited.

The lesion, uptake, and residence times seen in this study are in general concordance with our prior observations in patients with metastatic prostate cancer [18,20,21]. In prostate cancer patients, both WB and serum biologic half-times increased with increasing antibody mass. A significant difference in all intergroup values was noted, except for antibody masses of 50 and 100 mg. The current study involved escalation of antibody mass between cohorts as against within cohort, limiting the statistical power of the comparisons. However, the general trends of antibody mass dependency in terms of increased lesion uptake for higher mass-doses of antibody were similar across the studies.

Based on the increased lesion visualization in later scans compared to earlier time points, the optimal time for lesion detection, and therefore imaging, appears to be 5 to 7 days. This is similar to our prior observation in prostate cancer patients, wherein more lesions were seen at the last scan of imaging after each cycle of antibody administration [20]. In a more recent study with ^89^Zr-J591, we also saw more lesions at later time points and the optimal imaging time was determined to be 7 ± 1 day [25]. The larger number of lesions detected in images obtained at later time points is likely due to clearance of blood pool activity leading to higher tumor-to-background ratio at later time points, thus allowing for visualization of lesions with higher contrast. With an increasing number of infusions, it is possible that the saturation of antibody accumulation or pooling in normal organs occurs, e.g., in the liver, which may allow for more radiolabeled antibody targeting the lesions. Lesion targeting was seen in all tumor types studied, though due to the low number of patients included from each tumor type, no statistical differences in detection rates for specific tumor types could be derived.

The analysis shows potential of anti-PSMA imaging in patients with solid tumors. While the tumor types were not selected based on confirmation of PSMA staining, targeting of lesions was seen in all tumor types imaged in this study consistent with prior reports that showed PSMA expression in vasculature of these tumors [10,11,22]. PSMA-directed imaging and targeting of neovasculature in these tumors may play a potential role in developing novel therapies through direct use as a radioimmunotherapy agent or biomarker for assessment of efficacy of treatment, especially anti-angiogenic agents. Imaging time post-injection due to longer circulation time of the antibody and image quality related to single-photon-emitting radioisotope are both limitations of ^111^In-J591. Smaller anti-PSMA molecules, minibodies, or ligands directed to PSMA, preferably using PET imaging techniques, may be a more suitable approach in imaging these tumors. This approach also needs to be established in larger populations with a focus on specific tumor types.

## Conclusions

Antibody J591 imaging allowed visualization of lesions in solid tumors of diverse origin. The visualization of lesions is antibody mass-dependent and may be optimally seen at 20 mg antibody dose. The optimal scanning time appears to be 5 to 7 days post-injection. Further studies in larger subjects, preferably with smaller molecules directed to PSMA, are needed to further establish the role of PSMA-directed targeting of these tumors.
